# ”It Feels Like Being a Real Doctor:” The Virtual Family Approach in Medical Education

**DOI:** 10.15694/mep.2017.000187

**Published:** 2017-10-19

**Authors:** Don Robison, Senthil Rajasekaran, Norman Berman, Lisa Auerbach, David Henderson, Suzanne Rose, Lauren Mazzurco, Christine Matson

**Affiliations:** 1 Eastern Virginia Medical School; 2 Dartmouth University; 3 City College of New York; 4 University of Connecticut School of Medicine

**Keywords:** virtual family, case-based learning, longitudinal virtual family, social determinants of health, context of health

## Abstract

This article was migrated. The article was marked as recommended.

The majority of health outcomes are determined by social determinants of health (SDOH) while medical care is responsible for as little as 20% of health outcomes. This article is an introduction to the Virtual Family (VF) approach to case based instruction; a novel strategy for addressing SDOH in medical school. The VF theoretical framework is presented and practical considerations and challenges for implementation of the VF approach at three different medical schools are offered. VFs are defined as representations of families or social groups that are not real. “Virtual,” in this instance, refers to people or things that do not physically exist. The VF approach allows students and educators to adjust the “lens” of a case’s focus to view the relevant determinants. The VF approach is presented as an extension of the virtual patient approach. Theoretical support for the VF approach is argued drawing on principles from modeling and simulation, effective story design, establishing a sense of human presence, serious gaming, visual design, identity leveraging, and flow theory. Challenges and benefits of the approach are described. Measures of efficacy designed to match learning goals are proposed. The VF approach is presented as practical, accessible, economical, and potentially powerful.

## Introduction

The majority of health outcomes are determined by socioeconomic, educational, health behaviors, or environmental factors, while medical care is responsible for only 20% of outcomes ( Berman, Durning, Fischer, Huwendiek, & Triola, 2016). Undergraduate medical education has focused on this 20% of outcomes, but has addressed the other 80%, the social determinants of health, in a piecemeal way. An optimal presentation of these critical factors in health outcomes would be intentional, realistic, and embedded in authentic human experiences that engage students with complex challenges and opportunities. This article presents the theoretical framework for a novel concept for addressing these important topics developed at three different medical schools: the virtual family (VF) approach to case presentation in medical education.

Virtual families are defined as representations of families or social groups that are not real. There is no assumption of media delivery, learners may interact with a virtual family in a variety of ways. Examples include: an interactive simulation, a paper-based case, or an interaction with virtual medical records. Virtual, in this instance, refers to people or things that do not physically exist.

Virtual patients (VPs), the conceptual predecessor of VFs, have been used successfully in medical education in various settings ( Berman et al., 2011). Exemplified by intiatives such as Med U ^1^ VPs are more reliably accessible than real patients for teaching clinical reasoning, providing an assured and uniform patient experience across all learner populations ( Gesundheit et al., 2009), and can provide realistic learner interactions that result in the kind of deep learning for which effective medical education strives. This article focuses on the longitudinal VF approach employed at Eastern Virginia Medical School (EVMS) but also describes two other VF initiatives that focus on families and population health at Sopie Davis School of Biomedical Education/CUNY School of Medicine (SD/CUNY SOM), and the University of Connecticut School of Medicine (UCONN SOM).

At the heart of these three initiatives is the recognition that health outcomes most often flow from the broad context of a patient’s life and it is therefore imperative that we bring this context into our teaching and learning.

While VPs continue to be an effective complement to real and, in some instances, standardized patients, they still typically present an isolated interaction relatively limited in scope for addressing the multitiude of factors that affect health outcomes. One VP scenario can’t capture all the dynamics between the patient and caregivers, such as effects of low health literacy in both patient and caregivers, illness in a caregiver, loss of insurance in a dependent caregiver, or transition from military to civilian life. However as an integrated group of scenarios is created, the range of family dynamics can be systematically described, along with opportunities for caregiver support and facilitated access to community resources. With the growing ‘silver tsunami’ of aging patients with multiple complex, interacting constellations of illness, it is important to train future care providers to navigate these dynamics to more effectively collaborate with families in development of management plans.

## Previous work on virtual patients and impact on learning

For the purposes of this discussion we are considering VPs to be multimedia screen-based interactive patient scenarios ( Kononowicz, Zary, Edelbring, Corral, & Hege, 2015); this definition excludes other teaching methods that might be considered VPs such as simple case presentations, virtual patient games, high-fidelity software simulation, mannequin-based simulators and virtual standardized patients. It also excludes other forms of computer-based education such as digital slide presentations and educational video.

Berman et al. (2011) reported on collaborative development of VPs across multiple institutions making the task of covering broad curricular objectives more manageable and taking advantage of the ability to deliver VPs at scale, as suggested by Ellaway ( Ellaway, Poulton, Fors, McGee, & Albright, 2008). This development process led to a series of VPs primarily used in clinical clerkship education in the US ( Berman et al., 2011). A mixed-methods study of VP adoption based on the CLIPP program ( Schifferdecker, Berman, Fall, & Fischer, 2012) demonstrated that the program’s ability to fill gaps in exposure to core clinical problems, the use of a national curriculum, and development by clerkship directors were important factors leading to broad adoption of the program. VPs are used to achieve widely varied instructional goals including teaching core knowledge ( Sanders et al., 2007), clinical reasoning ( Garrett & Callear, 2001), communication skills ( Sijstermans, Jaspers, Bloemendaal, & Schoonderwaldt, 2007) and assessment ( Round, Conradi, & Poulton, 2009). Berman and colleagues showed that VP based learning can be effectively integrated into clinical education by coordinating their use with other learning activities, and reducing some lectures and textbook assignments ( Berman et al., 2009 Jul).

Despite the broad adoption of VPs, there is little evidence in the medical education literature that their use positively affects learning outcomes, a challenge shared by multiple educational interventions.

## Brief overview of the longitudinal virtual family approach at Eastern Virginia Medical School

**Virtual families fill a unique learning space.** The power of VPs in stimulating deep interactive learning experiences led to the conception of the VF approach. The VF approach is an extension of VPs. The difference between VPs and VFs-and the reason VFs are worth considering on their own merit-is that with VFs the case-based “lens” focuses more broadly so that the student view of a case includes not only the patient, but the patient’s immediate surroundings, context, and family. We describe this perspective as viewing the person-in-context (see Ford, 1992). This distinction can make a significant difference for learners in our effort to develop the clinical paradigm that a person’s health is the result of an environmental, social and biological system; and not just the biological system alone. In other words, based on an analysis of the VP literature and an understanding of the social determinants of health (SDOH), the VF approach is uniquely suited for teaching the SDOH.

This person-in-context effect could be accomplished through the employment of elaborate interactive simulation, but the impact of an elaborate simulation may also be achieved through the systematic application of principles from cognitive learning theory, serious gaming, story structure, and simulation design at a much more modest cost. Further, by EVMS incorporating an approach less dependent upon complex technology, a nearly ubiquitous employment throughout the undergraduate medical curriculum has been possible. This highlights another distinction between VPs and VFs: while VPs are, by definition, multi-media screen-based patient interactions, there is no assumption of media presentation with the VF approach, the emphasis is on presenting the patient in context. This approach of placing the patient in context also creates rich opportunities for integrating relevant socioeconomic, behavioral and environmental aspects.

### The longitudinal virtual family approach

The longitudinal VF approach employs a detailed medical story that unfolds over time for each family member, a detailed social history, simple but attractive visual representations of the family member and family, and carefully planned sequential interactions with the family and family members across the curriculum. Each case is treated as a repository of expertise (a classic use), a simulation (that is, a representation of the real world with which students may interact), and a story (providing a cohesive, human, and even emotional context).

As executed at EVMS, VF case presentations evolve in their complexity based on students’ developmental level. Early in the undergraduate medical curriculum, cases provide context for foundational science principles; later, cases elicit clinical logic and problem solving and stimulate group interaction. But in each case, students repeatedly engage with members of the same families. Students may meet Mr. Isaiah James (a VF member) as he is diagnosed with Type II Diabetes Mellitus at the beginning of M1 year when the learning objective is about tracing the biochemical and physiological aspects of glycemic control, see him several times through the course of the year as his condition progresses, and then actually diagnose complications of the disease related to poor glycemic control late in the M2 year when the learning objective is to develop a defensible differential diagnosis. In this way, students see the multitude of factors that determine health outcomes. So students will see the same patient over time, the patient’s presentation will vary according to developmental stage and learning objective. Mr James’ story also intersects with his mother needing increased coordination of care due to a life-limiting illness. Questions posed to students relate to clinical skills, but also challenge them to wrestle with the complexities of real-life patient scenarios.

## Placing virtual families in a theoretical framework

The longitudinal VF approach is based on theoretical constructs from cognitive learning theory, modeling and simulation, story development and plot, and serious gaming. Cognitive learning principles weave throughout the approach, so rather than describe them separately, they will be addressed within the discussion of the three main categories of research and theory below: the application of simulation principles to VFs, the application of principles of story development and plot, and the application of principles from serious gaming.

### Application of simulation principles to virtual families

A simulation is a model of the real world with which learners can interact. Viewing a case as a simulation provides unique opportunities for contextual learning, and also transforms a case from an intellectual exercise to practice-based exploratory learning.

**The novice-to-expert continuum.** Given their spectrum of expertise, novices and experts interact with--and evaluate--simulations differently ( Alessi & Trollip, 2005). A key simulation goal with novices is to limit cognitive load and not overwhelm the learner with complex tasks. A novice evaluates a simulation by the relative ease of use and overall simplicity. In contrast, an expert evaluates a simulation by how well it represents reality ( Alessi & Trollip, 2005).

**The zone of proximal development in virtual family cases.** In our approach to VFs, we carefully meter the complexity of cases applying Vygotsky’s zone of proximal development ( Vygotsky, 1978), defined as the difference between what a learner can do without help and what he or she can do with help. Optimally, cases should present at the higher level of the student’s current developmental stage. For teams, cases should be presented at a level of difficulty just past the individuals’ level of development, but so that the team together can solve the case while individual students likely could not.

In practice, this translates to cases early in the M1 year being tailored to provide a context for understanding foundational science. The case is “simple” as an example of practice, but provides an ideal medium for exploring a foundational concept in the context of practice. These can be paper-based cases. They present VF characters, but often in simple terms and with a single image. Late in the M2 year, on the other hand, cases present students with realistic decision points and push students towards authentic clinical decision making. The emphasis is less on foundational knowledge and more on effective medical practice.

**Model and simulation fidelity.** Alessi defines a *model* as the mathematics or logic that underlies a simulation, and a *simulation* as the model plus the interface that allows users to interact with it ( Alessi, 2000). Fidelity refers to the realism (usually understood in terms of detail) that the model or simulation presents. In the longitudinal VF approach, great care is taken in creating an underlying model that is sophisticated and accurate: a high-fidelity logic model. At EVMS, this model was created using a team of physicians, developing the medical history and progression for each of over 50 VF characters in detail. This level of attention to detail is critical because the health progression of each character over time must make sense to students. Great care must be taken to insure that the VF character medical histories are true-to-life given the family medical history and represented conditions.

**Figure 1.**
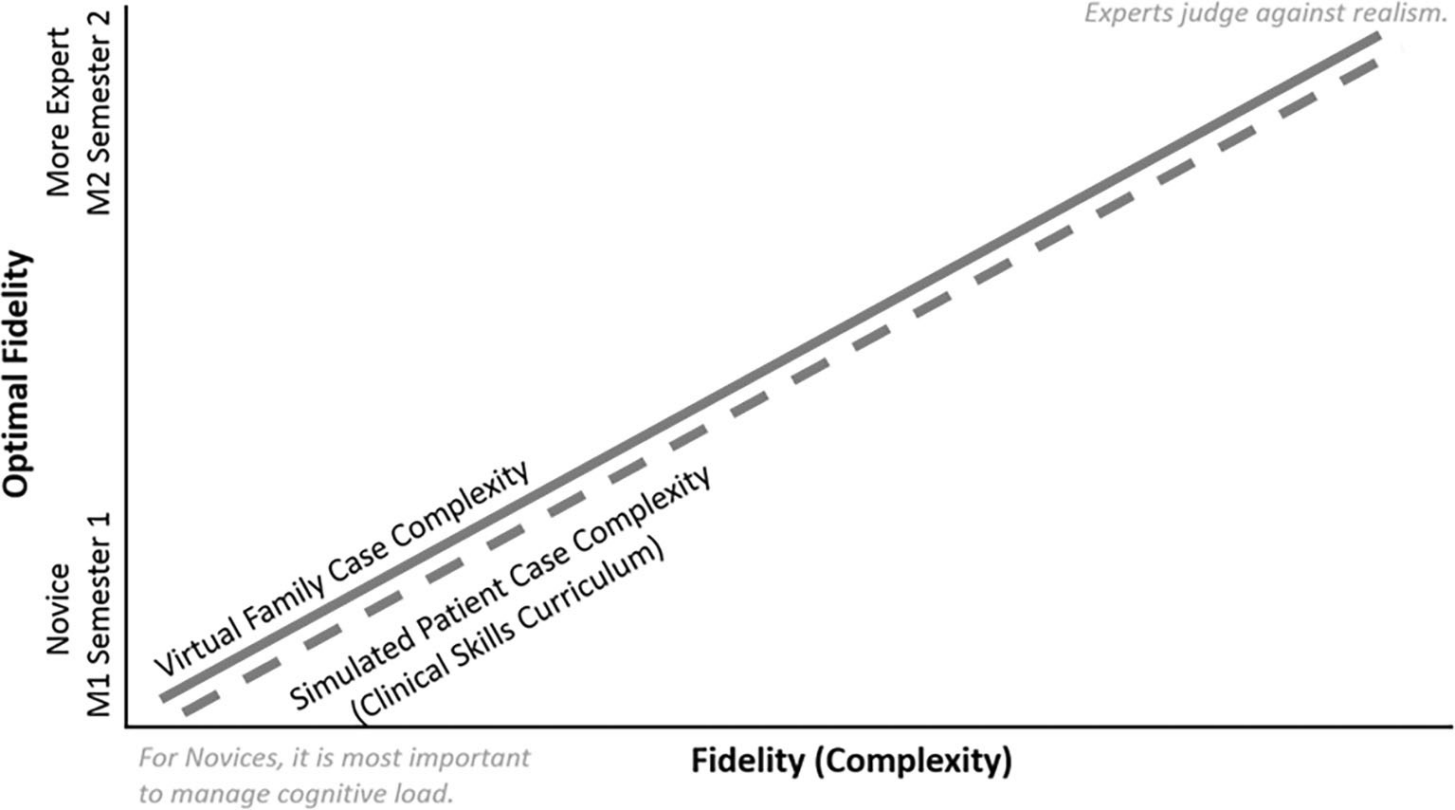
Optimal VF case complexity across the curriculum. Note that it parallels the optimal case complexity for practical skills instruction.

The VF’s do not exist in a vacuum, they are used in concert with other educational and simulation devices. It is important that VF case complexity be carefully coordinated with other education elements within the curriculum. For example, at EVMS VF case complexity is carefully paced with simulated patient complexity, though they are used to accomplish different objectives ( Figure 1).

### Facilitating understanding through story

Applying the principles of simulation is important, but our goal is to realize the positive effect of simulation without the cost or complexity of technologically sophisticated computer programming. Story provides a powerful and economical way to bring VFs to life with learners outside of technological dependency. Schank proposed that “we understand the world through story” ( Schank, 1990, p. 241). VFs naturally lend themselves to stories that have dynamic events, embedded emotions and thereby convey to students the significance or meaning of the illness to the patient.

**Personification and presence.** A sophisticated computer-based simulation can make a virtual character seem real through complex response algorithms. Users may experience a sense of presence, that is, the sense that a person is present. This is important in physician education as interview and diagnostic skills are essentially human skills. This is also important because people are social creatures, and long-term memory is enhanced with perceived social interaction ( Allmendinger, 2010; Hacker & Bol, 2004). This sense of presence, or the learner projecting personhood on a VF character, is personification.

**Figure 2.**
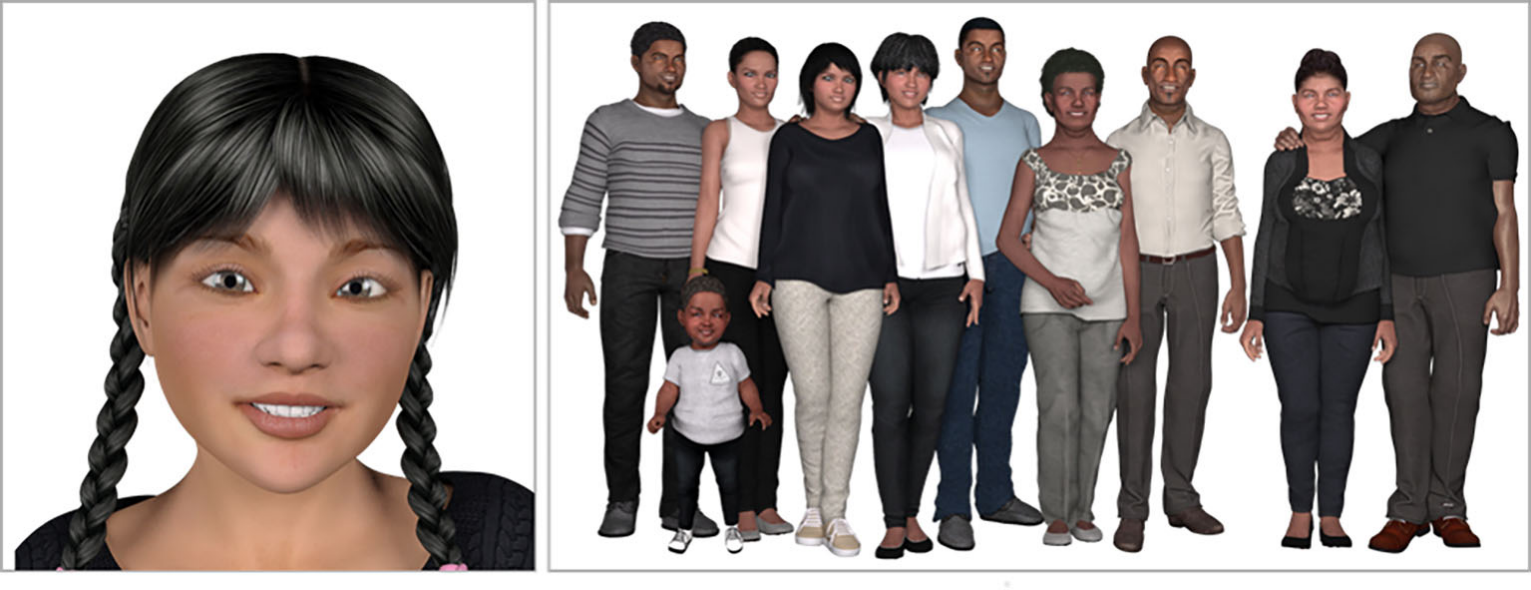
Personification may be partially achieved through the use of relatively simple images. In this case, 2D images were developed from low-cost 3D models. The image at left above is an example of a VF member portrait pose (one of 12 standard poses). The image at right above is the Johnson family, one of the VFs developed at EVMS.

Personification can also be achieved through story and simple visual representation for a fraction of the cost of computerized simulation. How people project humanness onto inanimate objects such as letters, texts, stories, or games is complex. The famous love letters between John and Abigail Adams demonstrate that each felt that the letter they were writing was a person-to-person communication as if the other were *present* in the room as they wrote ( Ellis, 2010). Text messages of today often carry the personal, even intimate, expression of people who perceive the other is personally present. An individual’s choice to project personhood on inanimate objects may be unconscious, and is likely the product of multiple variables (e.g., the availability of human-like prompts, technology characteristics, visual cues). Personification of VF characters is achieved through developed back-stories, realistic medical histories, and simple visual representations (see Appendix- Figure 2).

**Leveraging classic story structure.** Parrish demonstrated that learning experiences are most effective with learners when they mimic classic story structure ( Parrish, 2009). The elements of a story: setting and characters, problem, rising action, climax, falling action and denouement fit nicely with case presentation. In the case of VF cases, the end of a case is not an end to the story (usually), so the resolution must leave the door open for continued longitudinal relationship and care at a later date. Such an approach not only helps students make sense out of a case, but also accurately reflects the longitudinal nature of medical care.

### Applying principles from serious gaming to virtual families

Serious games are defined as “.. interactive computer-based game software.. developed with the intention to be more than entertainment” ( Ritterfield, Cody, & Vorderer, 2009, p. 4). Research in this arena provides principles relevant to VF case execution.

**Figure 3.**
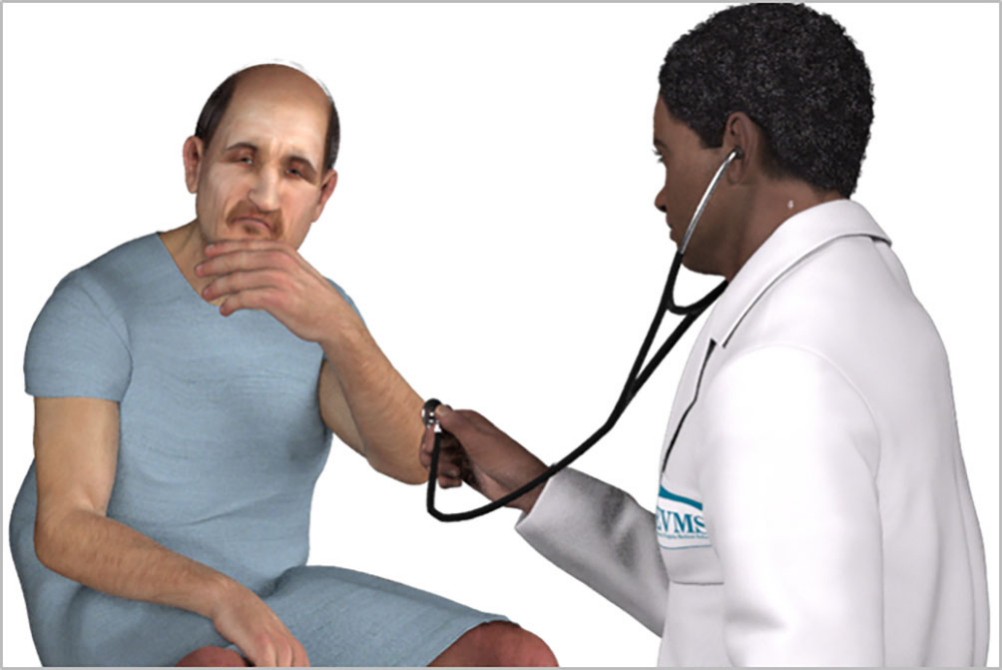
Using stills of easy-to-manipulate 3D models allows developers to create simple scenes that convey action. By not using photographically realistic images the negative effects of Mori’s ‘uncanny valley’ ( Mori, 2012) are avoided.

**Visual representation and design.** Borrowing from serious games, persons may be represented as either photographically realistic images or as relatively simple drawings. There is an entire category of popular online games, for example, that uses animated stick figures ( Poki.Com, 2017). In the longitudinal VF approach, visual representations are 2D images of 3D models. Each model is developed with 12 standard poses that may be used to create any number of scenes. A few of the poses are designed to allow a character to interact with other characters, completing the image of family. Characters are represented as high quality drawings even though the modeling software supports photographically realistic representation. This level of image quality helps avoid the negative effects of the uncanny valley ( Mori, 2012) described as a negative emotional response of people against human images that seem “almost” human but are not (Appendix- Figure 3). This level of image quality also allows for flexible staging of backdrops or virtual environments.

**Identity leveraging.** Erikson proposed that the search for identity is a primary motivator for adolescents and young adults ( Erikson, 1980). Game developers profit from this interest in identity when they create role-play games that allow players to personalize their avatars. Lee and Hoadley refer to this as “leveraging identity for fun” and observe that game players enjoy the activity ( Lee & Hoadley, 2007). By placing students in virtual contexts similar to their aspirations, students are able to live (even if virtually) in the future towards which they are working.

**Figure 4.**
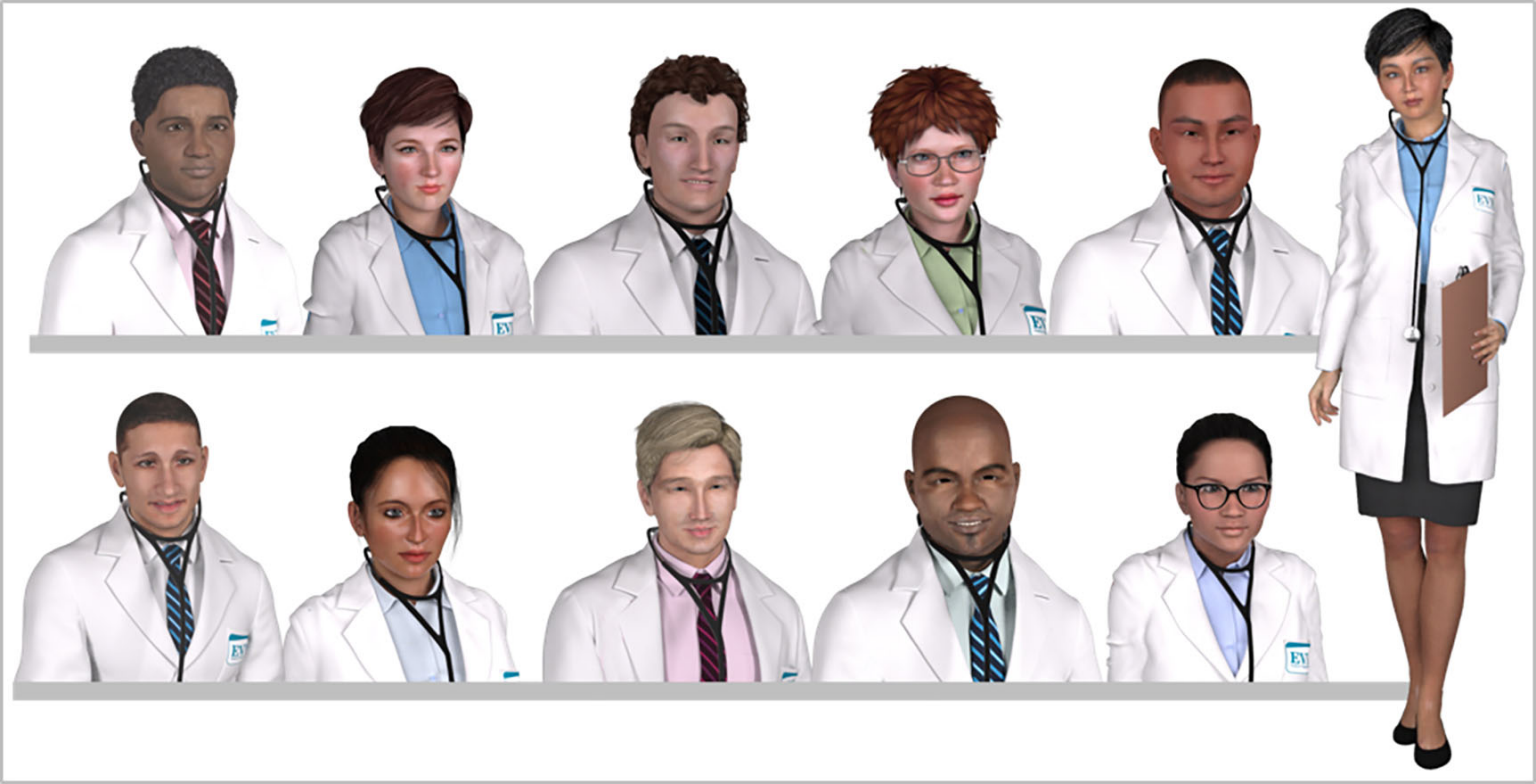
‘Young Physician’ images may be used two ways: First, in classroom-based team activities, diverse young physicians can be used to provide an aspirational picture of diversity in medicine; and second, in self-paced individual practice cases, students can choose their preferred image, in many cases enhancing the impact of identity leveraging.

**Fantasy.**In serious gaming fantasy is creating an environment that evokes mental images of “physical or social situations not actually present”( Malone & Lepper, 1988, p. 240). Students acting as physicians in the present to address authentic medical challenges is fantasy in this sense, and such fantasy has broad positive effects. First, it allows students to learn in a context similar to their later practice, enhancing far transfer of learned skills. Second, since cognitive structures are context-supported, learning is enhanced by placing it in a context similar to the performance context of the future. Third, placing students in their preferred future is both motivating in terms of desired identity, but actually helps them begin to form that valued professional identity. To facilitate this type of identity-related fantasy, several diverse ‘young physician’ models are used in cases (Appendix- Figure 4). For team experiences, the different young physician models are used in different cases; for individual practice experiences with online interactive cases, students may actually select the young physician character model of their choice, allowing them to immerse themselves in the role play of the case. Identity is a powerful motivational and developmental influence.

**Flow.** One lesson learned from VP cases is that it is important to keep the case moving. If a learner gets bogged down in details the experience will be negative. Incorporating that lesson, an important aspect of VF case employment is intentional monitoring of the learner flow experience. Flow is defined as “..the state in which people are so involved in an activity that nothing else seems to matter” ( Czikszentmihalyi, 1990, p.4). Serious gaming researchers refer to Czikszentmihalyi’s flow concept as a key measure of game experience quality. Developing cases with a primary goal of facilitating optimal flow experience helps insure an optimal learning experience, whether as a team or as an individual.

## Challenges and benefits with the virtual family approach

While the value of VP case use in undergraduate medical education has long-since been established, there is limited precedent for development and implementation of longitudinal VF use in undergraduate medical education. There are challenges intrinsic to this approach and implementation.

### Challenges intrinsic to the approach

The VFs have realistic backstories and dynamics, however they are not live patient encounters and this may generate frustration in eager learners. Similar to the unintended consequences of exploring SDOH described by Garg and his colleagues ( Garg, Boynton, & Dworkin, 2016), leaners may feel overwhelmed by social complexity, inability to relate due to personal experience or become disconnected due to feeling that many issues have no clear solution or path toward resolution. Expectations must be established from the outset that these VF cases are not expected to provide *the same* experience as interacting with a live patient. Rather, learning results from the *process.* Students are provided the opportunity to engage with application of the foundational sciences, grapple with effects of SDOH on health outcomes and bias, and develop clinical reasoning, problem solving and shared decision-making skills in a controlled setting. At the same time, empathetic responses to the illness experience will be developed, with greater understanding of the interplay between external barriers to healthy behaviors and internal motivation.

### Practical challenges to implementation

There are also challenges to the implementation of the longitudinal VF approach in medical education. Navigating the “best” approach to VF case integration into the curriculum is a challenge given the lack of precedent and competition for curricular time. Finding time for discussion of foundational or key concepts (e.g., Health System Sciences, SDOH, and defining chronic disease) and small group time for grappling with complex social dynamics and factors that affect health outcomes can be challenging, requiring the active collaboration of foundational science and clinical faculty. Similarly, availability of “expert” academic and community faculty to contribute or even participate in these group discussions may be limited as well.

Adequate representation of demographics should be considered and balanced with assignment of chronic conditions to prevent stereotypical bias toward various populations. Consistency in language and key aspects of expanded social history, such as employment status, insurance status, living arrangement, and material insecurity to name a few, should be established prior to case development. As a result, case writing and tracking of the evolution of the social history is time intensive. Consistency in the static and dynamic social history is critical to the authenticity of the character and family for case deployment over time. It is helpful to have an information management system to help develop and store cases and case-related media, and track objectives and content over time.

Available, skilled multidisciplinary faculty educators are necessary for case writing and vetting, as well as discussion guidance. Material needs include technology development or adaptation for writing, storing, and tracking cases over time. Finally, financial support is critical to support faculty time and effort (particularly clinical faculty) and support development of educational tools and technology.

Teaching faculty need to buy in to the innovation ( Schifferdecker et al., 2012). They need to adjust to new learning formats, content, knowledge and the time management related to these changes. Teaching faculty can feel that these changes cause more problems than solutions. By identifying opportunities for flexibility and creativity during implementation of the VF cases within their respective teaching modules, faculty may develop a needed sense of ownership.

In implementing this innovation learners need an effective means for providing constructive feedback, particularly if there have been significant changes in structure or time. Using the Plan-Do-Study-Act iterative four-stage problem solving model for process change can provide a framework in which iterative changes may be made ( Langley et al., 2009).

### Practical opportunities

The VF approach to case integration within medical education provides a unique opportunity for learners to develop an awareness of the factors, including medical and social, that affect patient and population health. There are innumerable opportunities for growth and practical application of this approach.

Most patients and families will experience, and likely succumb to a chronic condition or complications resulting from chronic disease. Identifying opportunities to integrate concepts of longitudinal chronic disease prevention and self-management as well as effects on family caregivers/family across the curriculum will better prepare phyhsicians to care for the patients and families they see everyday. As such, VF cases may be used in various settings including individual or online self-paced learning, small group discussion, and within traditional face-to-face classroom-based lecture. Cases can span not only across UME curriculum but into graduate medical education, academic and community-based physician continuing medical education (CME) as well as interprofessional education given the need for a team-based approach to care for patients with multiple complex conditions.

## Presenting authentic learning experiences at Sophie Davis School of Biomedical Education/CUNY School of Medicine

Motivated by the same SDOH learning objectives described above, VFs have been used in various ways at the Sophie Davis School of Biomedical Education/CUNY School of Medicine (SD/CUNY SOM). A new preclerkship curriculum uses VF characters in all Problem Based Learning experiences (PBLs) and Objective Structured Clinical Examinations (OSCEs). Longitudinal clinical experiences have been developed to allow students to follow real patients over years but logistically, it is difficult for students to experience the planned continuity. Even when experiences progress as planned, a student rarely follows a patient for more than two years. Using our VF patients we can “fast forward” through time so students can better observe the short and long term impact of disease.(MacNamara, Gainor, & Taylor) Multiple cases from a VF can allow students to deeply engage with the members of the family ( Sharp & Primrose, 2003). Students who were taught with family centered cases reported that the virtual cases created context and believable characters ( Marriott, Styles, & McDowell, 2012).

Students at SD/CUNY SOM are introduced to the family members of a multigenerational family in their preclerkship PBLs and formative OSCEs early in their first year of medical school. They practice communication skills with standardized patients who represent the members of this family. As the student continue to progress through our preclerkship curriculum, they will see these family members at different stages of their life and the patients, as in real life, will experience a variety of social and medical problems. The curriculum will culminate with a summative OSCE in which the patients will be members of a familiar family.

As VFs are developed, specific ethnic and socioeconomic characteristics are included. Race portrayal in lecture based traditional preclerkship curriculum often presents race as biology. This has been shown to increase student bias and may worsen inequities in the health care system ( Tsai, Ucik, Baldwin, Hasslinger, & George, 2016). This curriculum uses VFs to address these biases directly. Through the use of VFs, students learn to identify the impact of ethnicity and social determinants on health outcomes. They will also investigate interventions to mitigate negative impact of these factors on patients’ health and well-being.

## Use of virtual families in the MDelta Curriculum at the University of Connecticut School of Medicine

In August 2016, the University of Connecticut School of Medicine (UConn SOM) implemented the MDelta (Making a Difference in Education, Learning and Teaching) Curriculum with the incoming Class of 2020. This curriculum represents an 80% change in the pre-clerkship curriculum with elimination of lecture pedagogy in favor of team-based learning and with both a student-centered and patient-centered focus. There is a major emphasis in the Vertically Integrated Teams Aligned in Learning and Scholarship (VITALS) course on SDOH and disparities and all students will receive a certificate in public health from the Graduate School. More information about the MDelta Curriculum is available ( http://anyflip.com/onxj/rfnc/).

UConn SOM has utilized VFs in the curriculum for over ten years. VFs were initially embedded in the Family Medicine clerkship. Framing information in the context of clinical scenarios makes information more relevant and memorable; and being able to evaluate pathophysiologic problems in the context of psychosocial issues is not only interesting, but is imperative for personal and professional development.

**Figure 5.**
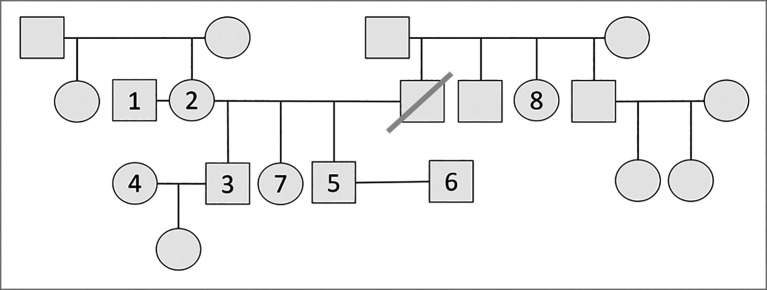
African American Family.

UConn SOM’s undergraduate medical education (UME) leadership team applied for the American Medical Association’s Accelerating Change funding, and was successful in receiving funding with the second cohort for a project implementing a teaching electronic medical record (tEMR). We have developed three VFs so that each unit in our Stage 1 course entitled: Case Oriented Essentials (COrE) is introduced by a member of the family. This Team Based Learning (TBL) course employs a ‘flipped classroom’ model with students preparing for each session of the TBL unit in advance. The course includes an integrated approach with over 50 units including: pathophysiology, molecular biology, genetics, and all discipline-based topics along with issues presented in a social context. Students will utilize the tEMR to be introduced to members of the VFs as it pertains to the unit under discussion. Our plan is to further develop the three established families (see example, Appendix- Figure 5) in order to explore other areas of diversity. The plan includes linking objectives in the basic sciences, clinical medicine and social sciences to each family member. The longterm plan is that the patients will also be revisited over the four years of the curriculum in other courses and venues.

1. Mark Robinson - 54 yo postal worker. High School Grad. Obese, Sedentary
2. Connie Carter Robinson - 53 yo CNA. Associates Degree. Obese, Sedentary
3. Marvin Carter - 28 yo unemployed roofer. History of a work-related injury 2 yrs ago. Since then he has been living with his girlfriend, Sonia and their 14 mo old daughter. High School Grad, started but dropped out of community college.
4. Sonia Camacho - 22 yo unmarried Latina, part-time Stop and Shop employee. High School Grad.
5. Martin Carter - 30 yo employed as actuary at the Hartford. He is gay and lives with his partner of the past 5 years, Samuel. College Grad. They are in the process of adopting a toddler with asthma.
6. Samuel Ross - 28 yo Caucasion partner of Martin. College Grad. Engineer at Pratt and Whitney.
7. Camille Carter - 32 yo. High School Grad. Hx of poorly controlled bipolar disorder. She lives in public housing and is on disability due to bipolar disorder and receives SSI.
8. Carol Carter - 60 yo with longstanding history of schizoaffective disorder. Lives with parents.

## Proposed measures of efficacy

A meta-analysis of the learning effects of VPs concluded that there are large positive learning gains from VPs compared to no intervention, but that comparison to other educational interventions shows small gains ( Cook, Erwin, & Triola, 2010). Cook and Triola suggested, based on learning theory and a review of the literature, that VPs may be particularly effective for teaching and assessing clinical reasoning.

As the increased use of VFs may be contemplated in medical school curricula, it is critical that the construct be clearly defined in measurable terms, and that the construct being measured should relate to the problem being solved by VFs. With VFs, the unique learning goals relate to a) an improved sense of the impact of SDOH on health and disease, b) a realistic view of the longitudinal nature of patient care, c) study of foundational science concepts in the context of patient care, d) clinical reasoning, e) understanding the psychosocial factors affecting health outcomes, and f) improved student engagement in cases. Table 1 presents proposed efficacy measures for VFs.

**Table 1.**
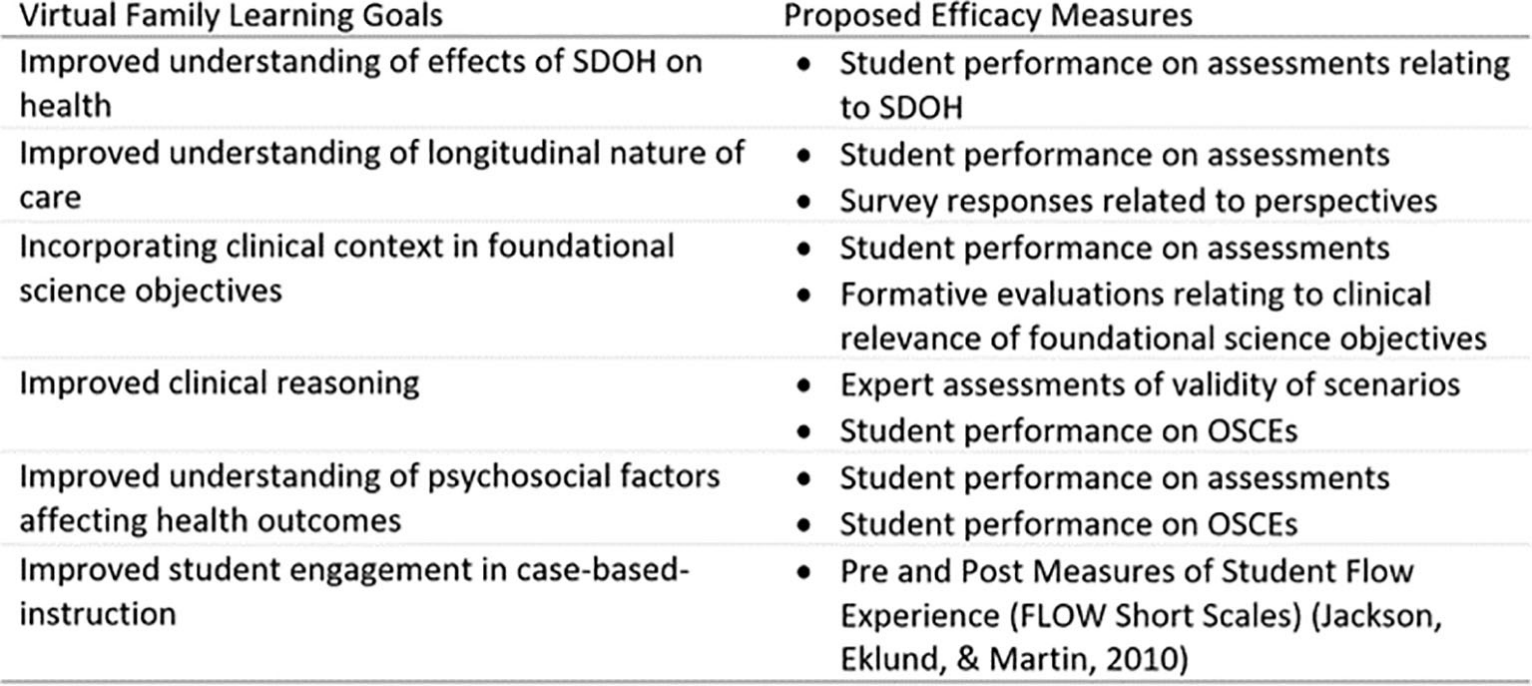
VF Goals and Proposed Efficacy Measures

## Conclusions

The success of VPs in facilitating deep learning led directly to the conception of the VF approach to case based medical education. VFs are an extension of the virtual patient concept, and overlap virtual patient execution in many dimensions. The difference is that VFs focus on the person-in-context and therefore provide an exceptional vehicle for engaging learners with the varied determinants of health in a structured, inclusive way.

The VF approach does not assume a medium, and so three different versions of the approach were reported here. At the heart of these three initiatives is the realization that health outcomes result from the context of a patient’s life interacting with the patients’ inherited propensities and so each initiative works to bring that patient context into the teaching and learning experience. Could the same thing be accomplished through an elaborate virtual patient case? The answer is clearly yes: there is much overlap in the two concepts. The essential element of the VF approach is not the media presentation, it is the contextual presentation. It is this focus on context rather than media that provides the greatest opportunity for the VF approach: although the complexity requires significant resources for development, the model’s implementation is financially attainable. In fact, at EVMS, VFs provide a sort of ubiquitous simulation environment for longitudinal case-based learning that would not have been feasible with high-end computerized simulations.

To understand the context of patient health fully-to effectively treat patients in view of the full picture of health-it is essential that medical students learn the skills of medical practice in scenarios that imitate their patients’ lives. Virtual families provide an excellenct vehicle for such learning.

## Take Home Messages

• Cases involving characters and families that students see longitudinally across the medical school curriculum matches authentic medical practice.
• Health outcomes occur in the context of family or immediate ‘others.’ Routinely presenting cases in the context of family mimics life.
• Relatively simple tactics (story, intentional image design, careful planning of disease progressions) may significantly enhance student perceptions of personhood in case based instruction.
• We can teach the complexities of the social determinants of health in technologically simple ways.
• The VF approach is media-neutral, the focus is on presenting the patient in an authentic social and environmental context.

To understand the context of patient health fully-to effectively treat patients in view of the full picture of health-it is essential that medical students learn the skills of medical practice in scenarios that imitate their patients’ lives. Virtual families provide an excellenct vehicle for such learning.

## Notes On Contributors

**Don Robison, Ph.D., CPT** is an instructional designer and the Director of Service Learning at Eastern Virginia Medical School.

**Senthil Rajasekaran, M.D.** is the Associate Dean of Academic Affairs at Eastern Virginia Medical School and leads the CareForward Curriculum Reform there.

**Norman Berman, M.D.** is a pediatric cardiologist, an educator at Dartmouth University, and a leader in maturing the Virtual Patient approach in medical education.

**Lisa Auerbach, M.D., MHPE** is an internist and Course Director of Organ Systems and Introduction to Clinical Medicine at City College of New York.

**David Henderson, M.D.,** is a family physician and educator who serves as Associate Dean for Student Affairs and the Associate Dean for Multicultural and Community Affairs at the University of Connecticut School of Medicine.

**Suzanne Rose, M.D., MSEd** is the Senior Associate Dean for Education at the University of Connecticut School of Medicine.

**Lauren Mazzurco, D.O.** specializes in Geriatric Medicine and is the Director for Case-Based Learning at Eastern Virginia Medical School.

**Christine Matson, M.D.** is a family physician who served as the Glenn R. Mitchell Chair of Generalist Medicine and Chair of the Department of ‌Family and Community Medicine at Eastern Virginia Medical School.
