# Supplementary material for: ”It Feels Like Being a Real Doctor:” The Virtual Family Approach in Medical Education
Source: MedEdPublish (2016). 2017 Oct 19;6:187. [Version 1] doi: 10.15694/mep.2017.000187 (PMC10885308; doi:10.15694/mep.2017.000187)
Supplement: Peer review history

# Reviewer report: Reviewer response for version 1

**DOI:** 10.21956/mep.19375.r28372

**Published:** 2018-10-24

**Authors:** Meghana Sudhir

**Affiliations:** 1 Mohammed Bin Rashid University of Medicine and Health Sciences

This review has been migrated. The reviewer awarded 4 stars out of 5 Thank you for sharing this longitudinal virtual family approach. The paper clearly explains the virtual family approach and how it is implemented. The challenges and benefits of this approach is also well addressed. Transfer of this approach to other Universities is also shared in this paper. The paper highlights the cost effectiveness of this approach as well as a full picture of health in the family. This approach is recommended in all health professions education program.

Reviewer Expertise:

NA

No decision status is available

# Reviewer report: Reviewer response for version 1

**DOI:** 10.21956/mep.19375.r28370

**Published:** 2017-10-24

**Authors:** P Ravi Shankar

**Affiliations:** 1 American International Medical University

This review has been migrated. The reviewer awarded 4 stars out of 5 I enjoyed reading this paper about the use of virtual families in medical education. With the increasing presence of technology and decreasing cost, virtual families (VF) could be a feasible option even in resource constrained settings. I do agree with the authors that the social determinants of health do not receive the attention they deserve. Most of the curricular time and effort is devoted to the biological determinants of health. The authors have provided a detailed and sequential description of VF in the manuscript. Longitudinal follow up of patients and families is important and VF can offer an approach toward doing so. I especially liked the section on ‘Placing virtual families in a theoretical framework’. In many developing countries real patients and families continue to be accessible. However, increasing development and higher level of patient awareness and the increasing complexity of modern life could constrain access. Virtual patients and virtual families can become a cost-effective alternative and as the authors have demonstrated teams at different medical schools could collaborate on developing virtual alternatives. The manuscript is well written and reads well, however, there are some minor spelling errors which can be corrected.

Reviewer Expertise:

NA

No decision status is available

# Reviewer report: Reviewer response for version 1

**DOI:** 10.21956/mep.19375.r28371

**Published:** 2017-10-20

**Authors:** Subha Ramani

**Affiliations:** 1 Harvard Medical School, Brigham and Women's Hospital

This review has been migrated. The reviewer awarded 4 stars out of 5 I enjoyed reading this paper. The authors have zoomed in on a very important initiative that targets learning about social determinants of health. They have adroitly pointed out how little time medical schools spend on educating students on these topics that contribute to 80% of patient care outcomes. They have creatively expanded the use of virtual patient- based learning to virtual-family based learning. It is true that today students can no longer be exposed to a variety of essential patient care experiences and scenarios without the use of virtual patients, simulations etc. Making these experiences as realistic as possible would greatly add to clinical learning. Their reasoning is very sound when they suggest that virtual patients in isolation do not represent real-life clinical experiences and virtual families is a great adaptation to emphasize social determinants of health. The authors trace the evolution of the use of virtual patients in clinical teaching and lead in to their approach on the need for and use of virtual families. I like the description of the cases and challenges intrinsic to the approach and related to implementation. I have a few constructive comments: In the introduction, I might have moved the paragraph on virtual patients above the paragraph that discussed virtual families to enable a more logical progression towards their study. I like the 3 categories under the theoretical framework overall, but I feel the authors may have tried to tackle too many theories discussed under each section. It might have been easier for readers if the authors focused fewer but in greater depth. This section could add to the cognitive load of readers. Finally, I would like to have seen a table to compare and contrast different approaches to this curriculum development and implementation and challenges which could make the project more accessible to other educators.The paper will be most valuable to educators working in the areas of simulation or virtual patient / family. There are also several messages that would be useful to all health professions educators who should all be focusing on social determinants of health.

Reviewer Expertise:

NA

No decision status is available

# Reviewer report: Reviewer response for version 1

**DOI:** 10.21956/mep.19375.r28368

**Published:** 2017-10-19

**Authors:** Julie Hunt

**Affiliations:** 1 Lincoln Memorial University

This review has been migrated. The reviewer awarded 4 stars out of 5 This article describes the concept of virtual family (VF) use in educating medical students to appreciate and evaluate the social determinants of health. Similar to the use of virtual patients, VF is a richer, expanded dynamic. The authors point out a few keys to attaining maximum impact, including allowing cases to unfold in real time (that is, over months and years). The authors provide a brief but helpful explanation of the theoretical frameworks that were used in VF exercises. I agree with the authors' call for additional research into the efficacy of the VF teaching method.

Reviewer Expertise:

NA

No decision status is available

# Reviewer report: Reviewer response for version 1

**DOI:** 10.21956/mep.19375.r28367

**Published:** 2017-10-19

**Authors:** Trevor Gibbs

**Affiliations:** 1 AMEE

This review has been migrated. The reviewer awarded 3 stars out of 5 An interesting paper about a novel approach to using an holistic virtual family to stimulate learning- a virtual family approach to problem-based learning. I thought that the early part of the paper was important in bringing in the theories behind the project- very similar to PBL and very helpful to anyone moving into the area.I think that the paper could have been more helpful and perhaps more practical if the authors had paid more attention to the major issues with case-development, and that is linking the case to specific learning outcomes, a problem for many who structure competency-based curricula. The paper because much had been spoken of the background to the project seemed to stop short of this important issue

Reviewer Expertise:

NA

No decision status is available

# Reviewer report: Reviewer response for version 1

**DOI:** 10.21956/mep.19375.r28369

**Published:** 2017-10-19

**Authors:** Ronald M Harden

**Affiliations:** 1 AMEE

This review has been migrated. The reviewer awarded 4 stars out of 5 I strongly recommend this paper. as it highlights some possibilities in using a virtual family as a valuable resource in the curriculum. Steve Smith from Brown University first drew my attention to the approach many years ago and I have been disappointed that more use has not been made of it. . As described the student working with the virtual family takes a necessary broader perspective of patient care and the importance of the social environment - valuable to integrate behavioural sciences in the early years of the curriculum. The fact that the patient and family evolve over time can also be associated with important messages. I was pleased to see that the paper argues that the patient and the family can be presented with different media and focusses on education issues such as realism. I was left wanting to know more about the experiences in the schools but this is not a bad thing. Congratulations on drawing to our attention a powerful tool

Reviewer Expertise:

NA

No decision status is available
